# New Diagnostic Model for Clinically Significant Prostate Cancer in Biopsy-Naïve Men With PIRADS 3

**DOI:** 10.3389/fonc.2022.908956

**Published:** 2022-07-04

**Authors:** Chen Huang, Feng Qiu, Di Jin, Xuedong Wei, Zongxin Chen, Ximing Wang, Xiaojun Zhao, Linchuan Guo, Jinxian Pu, Jianquan Hou, Yuhua Huang

**Affiliations:** ^1^ Department of Urology, The First Affiliated Hospital of Soochow University, Suzhou, China; ^2^ Department of Anesthesiology, The First Affiliated Hospital of Soochow University, Suzhou, China; ^3^ Department of Radiology, The First Affiliated Hospital of Soochow University, Suzhou, China; ^4^ Department of Pathology, The First Affiliated Hospital of Soochow University, Suzhou, China

**Keywords:** adjusted prostate-specific antigen density of peripheral zone, biopsy, diagnosis, PIRADS, prostate cancer

## Abstract

**Purpose:**

The aim of this study was to explore a new model of clinical decision-making to predict the occurrence of clinically significant prostate cancer (csPCa).

**Patients and Methods:**

The demographic and clinical characteristics of 152 patients were recorded. Prostate-specific antigen (PSA), PSA density (PSAD), adjusted PSAD of peripheral zone (aPSADPZ), and peripheral zone volume ratio (PZ ratio) were calculated and subjected to receiver operating characteristic (ROC) curve analysis. The calibration and discrimination abilities of new nomograms were verified with calibration curve and area under the ROC curve (AUC). The clinical benefits of these models were evaluated by decision curve analysis and clinical impact curves.

**Results:**

The AUCs of PSA, PSAD, aPSADPZ, and PZ ratio were 0.521, 0.645, 0.745, and 0.717 for prostate cancer (PCa) diagnosis, while the corresponding values were 0.590, 0.678, 0.780, and 0.731 for csPCa diagnosis, respectively. All nomograms displayed higher net benefit and better overall calibration than the scenarios for predicting the occurrence of csPCa. The new model significantly improved the diagnostic accuracy of csPCa (0.865 vs. 0.741, *p* = 0.0284) compared with the base model. In addition, the new model was better than the base model for predicting csPCa in the low or medium probability while the number of patients with csPCa predicted by the new model was in good agreement with the actual number of patients with csPCa in the high-risk threshold.

**Conclusions:**

This study demonstrates that aPSADPZ has a higher predictive accuracy for csPCa diagnosis than the conventional indicators. Including aPSADPZ, PZ ratio, and age can improve csPCa diagnosis and avoid unnecessary biopsies.

## Introduction

Prostate cancer (PCa) has become the second most common male cancer that affects approximately 375,000 men/year worldwide (1). The incidence of PCa has risen dramatically in recent years, especially in China, where it ranks second among male tumors (2). The multiparameter magnetic resonance imaging (mpMRI) has been increasingly used to diagnose patients with PCa in recent years (3, 4). In 2012, the European Society of Urogenital Radiology (ESUR) established a series of guidelines for the interpretation of mpMRI images using a structured reporting scheme called Prostate Imaging Reporting and Data System (PIRADS) (5). In 2015, the American College of Radiologists, EUSR, and the AdMeTech Foundation improved and updated PIRADS to version 2 (PIRADS V2) (6). In 2019, they upgraded PIRADS V2 to V2.1 (7). The PIRADS consists of five levels, ranging from 1 (clinically significant cancer is highly unlikely to present) to 5 (clinically significant cancer is highly likely to present). Of these, a score of 3 means the presence of clinically significant cancer is equivocal (8). In previous reports (9–11), the detection rate of PCa in PIRADS 3 lesions ranged from 11% to 33.3%, and the rate of clinically significant PCa (csPCa) ranged from 4.2% to 12%. Therefore, we are more concerned about whether there are other indicators that can help us detect more PCa and avoid unnecessary biopsy in patients with PIRADS v2 category 3 (PIRADS 3). In this paper, we established a new model to increase the detection rates of PCa and csPCa, and compared its diagnostic performance with the conventional model.

## Patients and Methods

### Ethical Approval

All patients were counseled about the risks of the procedure, and then, they signed a consent form that included permission to use their clinical data for research. Ethical approval was obtained from the Institutional Review Board of The First Affiliated Hospital of Soochow University.

### Patient Recruitment

**Figure 1:**
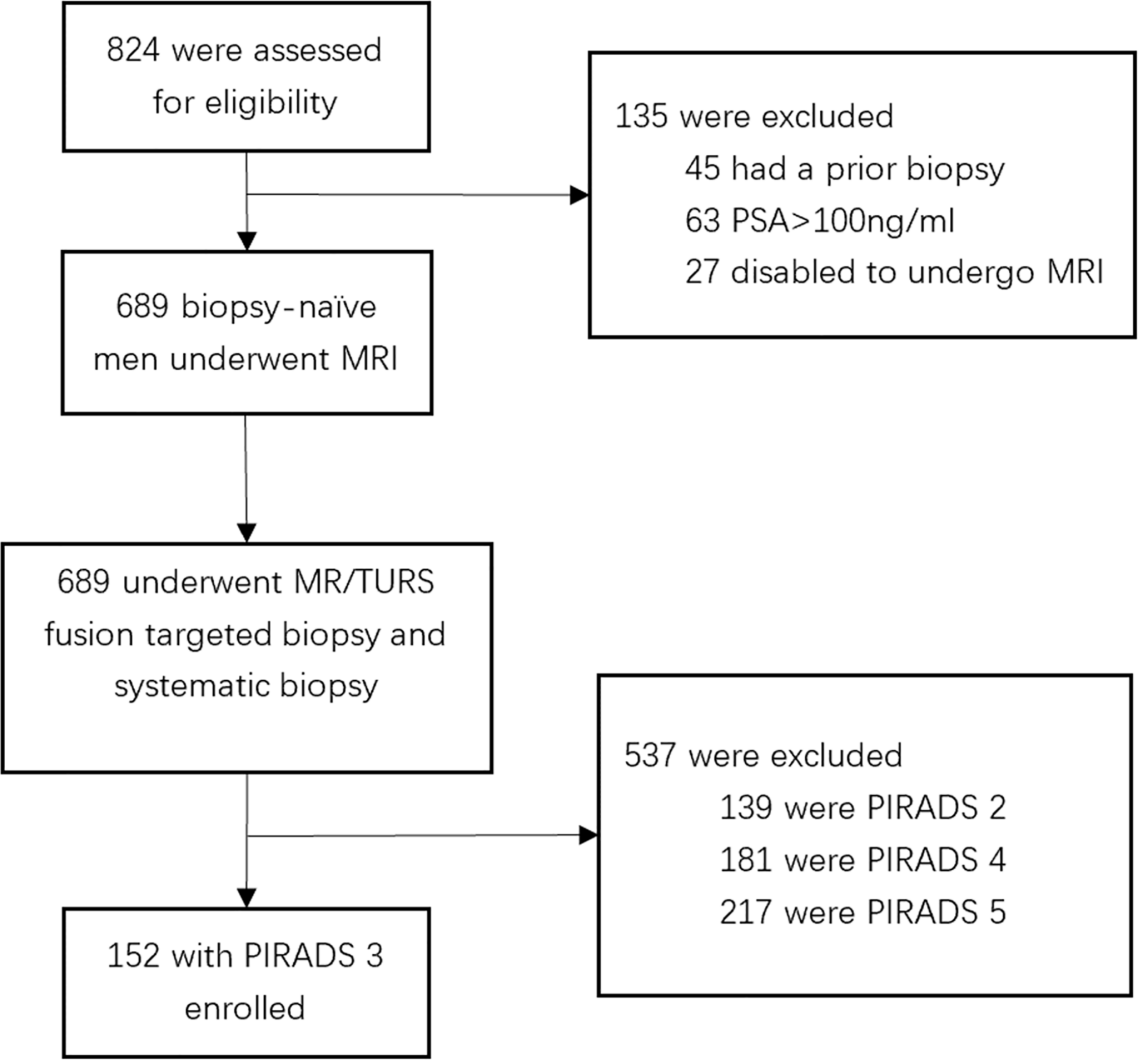
Flowchart for study inclusion among biopsy-naïve men with PIRADS 3 with clinical suspicion for prostate cancer.

In this retrospective cohort study, patients with PCa were recruited at The First Affiliated Hospital of Soochow University (Suzhou, China) from July 2016 to June 2020. A total of 824 male patients presented to our institution for prostate biopsy (PB). Of these patients, 45 had prior treatment, 63 had prostate-specific antigen (PSA) > 100 ng ml^−1^, 27 were not able to undergo MRI examination, and the remaining 689 received a transperineal PB. Among them,139 with PIRADS 2, 181 with PIRADS 4, and 217 with PIRADS 5 were excluded, and 152 with PIRADS 3 were included in this study. The patient selection flowchart is shown in **Figure 1**.

### MRI Acquisition

All patients were subjected to a 3-T magnetic resonance (MR) scanner (MAGNETOM Skyra, Siemens Healthineers, Erlangen, Germany). The 18-channel body and standard spine array coils were employed for signal reception. The transverse T1-weighted turbo spin-echo (TSE) images, as well as the transverse, coronal, and sagittal T2-weighted TSE images of the prostate and seminal vesicles were acquired. The apparent diffusion coefficient was obtained from diffusion-weighted imaging (DWI) and was calculated using a 2-dimensional echo planar imaging sequence with multiple b-value acquisitions (0, 100 s mm^−2^, 800 s mm^−2^, 1,000 s mm^−2^, and 1,500 s mm^−2^), with diffusion-sensitizing gradients applied along the *x*-, *y*-, and *z*-axes. Dynamic contrast-enhanced (DCE) imaging was conducted through a 3-dimensional T1-weighted gradient-echo volumetric interpolated breath-hold examination, and was in the same plane as the 3D T2W sequence. Then, an intravenous contrast agent (Medtron AG, Saarbruecken, Germany) was administered at 1 ml kg^−1^ body weight and 2.5 ml s^−1^ injection rate. The MR Tissue4D software (Syngo. *via* VA20B; Siemens Healthineers) was used to construct perfusion curves. The details of the imaging protocol are shown in **Supplementary Table 1**.

### Prostate Biopsy and Pathology Analysis

**Figure 2:**
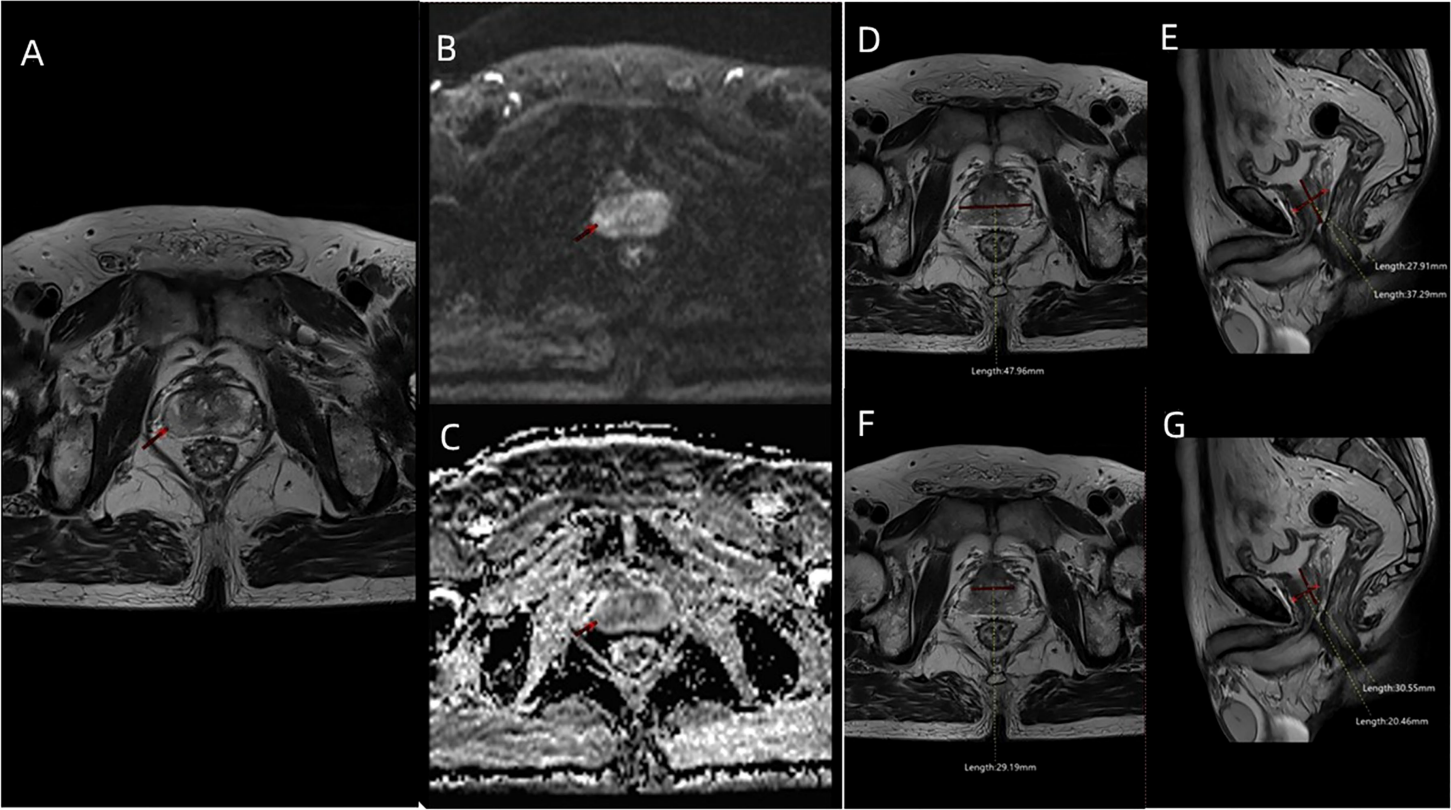
The MRI images including **(A)** T2WI sequence, **(B)** DWI sequence, and **(C)** ADC sequence of a patient (age: 66 years; PSA: 7.8 ng/ml; f/tPSA: 0.138; ISUP GG: 2) with PIRADS 3 ROI in the right lateral posterior of peripheral zone. **(D)** Maximum transverse diameter of prostate (47.96 mm) measured on axial T2WI, and **(E)** maximum longitudinal diameter (37.29 mm) and maximum AP diameter (27.91 mm) of prostate measured on mid-sagittal T2WI. **(F)** Maximum transverse diameter of transitional zone (29.19 mm) measured on axial T2WI, and **(G)** maximum longitudinal diameter (30.55 mm) and maximum AP diameter (20.46 mm) of transitional zone measured on mid-sagittal T2WI.

Transperineal prostate targeted biopsy (TB) and systematic biopsy (SB) were performed on all patients. During TB, the DICOM data of mpMRI images (**Figures 2A–C**), including T2WI, DWI, apparent diffusion coefficient (ADC), and DCE, were imported into the Real-time Virtual Sonogra (RVS) ultrasonography host (Preirus, Hitachi, Japan), and the target lesion was marked as region of interest (ROI). Through RVS, the ROI marked on MRI images was displayed in real time on the ultrasonography images. Ultrasonography and MRI images were matched by sagittal and axial anatomical markers, such as urethral orifices and small prostate cysts. Following these steps, the urologist performed the TB, and each ROI was executed on 2-core biopsy. After completion of TB, the RVS was turned off and the same urologist continued to perform SB. All specimens were fixed in 10% formalin and subjected to pathological analysis. The csPCa was defined as a single biopsy core with a Gleason score of 3 + 4 or above [International Society of Urological Pathology (ISUP) grade group (GG) >1] as described previously (9).

### Patient Characteristics

The patients’ age, pre-biopsy PSA, free/total PSA(f/tPSA), and pathological features were included in the study. The included MRI characteristics were PIRADS scores, prostate volume (PV) [prostate volume (PV) = 0.52 × height × length × width] (**Figures 2D, E**), PSAD (PSAD = PSA/PV), transitional zone volume (TZV) [TZV = 0.52 × height (TZ) × length (TZ) × width (TZ)] (**Figures 2F, G**), peripheral zone volume (PZV) (PZV = PV − TZV), PZ ratio (PZ ratio = PZV/PV), PSADPZ (PSADPZ = PSA/PZV), and adjusted PSADPZ (aPSADPZ = PSAD × PZ ratio). Each patient was graded according to PIRADS V2 by the same radiologist who graded more than 500 prostate MRI readings. The biopsy cores were examined by a dedicated pathologist.

### Statistical Analysis

Categorical and continuous variables were analyzed using the Pearson’s chi-squared test and Mann–Whitney *U* test, respectively. Binary logistic regression was used to calculate the odds ratios of each predictive factors. The predictive models were constructed as follows. First, univariate regression analysis was performed to evaluate the power of each parameter in diagnosing PCa and csPCa. Next, the variables with *p*-value < 0.05 in the univariate analysis were further analyzed by multivariate logistic regression models using the procedure of enter selection method. The multivariate regression coefficients were then used to construct nomograms. From multivariable binary logistic analysis, the following predictive models were built to predict the occurrence of PCa and csPCa: the base model included clinical factors such as PSA, f/tPSA, and PSAD, while the new model included age, PZ ratio, and aPSADPZ. The calibration and discrimination abilities of these models were evaluated using the calibration curve (1,000 bootstrap resamples) and area under the receiver operating characteristic (ROC) curve (AUC), respectively. The nomograms were also validated using an internal validation cohort (1,000 bootstrap resamples). The clinical benefits of these models were determined by decision curve analysis (DCA). In this case, we focused on 10%–40%, in which clinical decision-making is particularly difficult. The AUCs of both models were compared using methods described previously (12). In DCAs, the horizontal line along the *x*-axis indicated that all patients developed PCa and csPCa. The nomogram, calibration plots, and DCA were constructed by R x64 4.0.2 (http://www.r-project.org, last accessed date: 03/10/2022 18:10:01). Other statistical tests were conducted with SPSS v22.0 (IBM Corp, Armonk, NY, USA) and MedCalc v18.2.1 (MedCalc Software, Belgium). All reported *p*-values were two-sided and the level of statistical significance was set at *p* < 0.05.

## Results

### Demographic and Clinical Characteristics

Overall, 19.1% of patients (29/152) had histologically confirmed PCa, while 11.8% (18/152) had histologically confirmed csPCa. The clinical data of all patients are summarized in **Table 1**. PCa patients had significantly higher PSAD, PZ ratio, and aPSADPZ, and lower f/tPSA, PV, and TZV, compared to patients with benign disease. Similar results were observed for the differences in these parameters between the csPCa group and the benign or clinically insignificant prostate cancer (isPCa) group.

**Table 1:** Patient demographics and the correlation with biopsy results.

Characteristic	PCa (*n* = 29)	Benign (*n* = 123)	*Z*	*p*	csPCa (*n* = 18)	isPCa or benign (*n* = 134)	*Z*	*p*
Age (years), median (IQR)	68.0 (60.5–73.5)	65.0 (61.0–70.0)	−1.469	0.142	69.5 (64.0–76.3)	65.5 (61.0–70.0)	–2.192	0.028
PSA (ng ml^−1^), median (IQR)	7.90 (6.00–13.66)	7.84 (5.87–12.26)	–0.347	0.729	10.39 (7.26–16.05)	7.67 (5.92–12.17)	–1.235	0.217
f/tPSA, median (IQR)	0.120 (0.084–0.157)	0.160 (0.117–0.218)	–2.978	<0.01	0.100 (0.071–0.151)	0.15 7 (0.111–0.212)	–3.165	<0.01
PV (ml), median (IQR)	36.1 (25.7–55.5)	51.0 (39.1–69.2)	–2.884	<0.01	36.5 (25.1–57.1)	50.0 (37.5–68.3)	–2.138	0.032
PSAD (ng ml^−2^), median (IQR)	0.229 (0.136–0.392)	0.171 (0.111–0.246)	–2.427	0.015	0.251 (0.143–0.445)	0.176 (0.112–0.248)	–2.446	0.014
TZV (ml), median (IQR)	15.9 (10.0–26.6)	28.5 (18.7–45.7)	–3.833	<0.01	14.4 (10.4–25.9)	27.2 (18.3–45.1)	–3.165	<0.01
PZV (ml), median (IQR)	20.8 (13.8–27.0)	21.2 (14.1–29.6)	–0.288	0.773	22.5 (13.6–29.5)	21.0 (14.0–29.0)	–0.319	0.749
PZ ratio, median (IQR)	0.542 (0.440–0.641)	0.421 (0.301–0.533)	–3.632	<0.01	0.570 (0.456–0.675)	0.428 (0.301–0.534)	–3.182	<0.01
PSADPZ (ng ml^−2^), median (IQR)	0.459 (0.251–0.671)	0.413 (0.276–0.684)	–0.302	0.762	0.528 (0.220–0.767)	0.414 (0.277–0.647)	–0.661	0.508
aPSADPZ (ng ml^−2^), median (IQR)	0.119 (0.082–0.217)	0.059 (0.040–0.108)	–4.096	<0.01	0.249 (0.142–0.441)	0.064 (0.040–0.104)	–3.855	<0.01

PCa, prostate cancer; csPCa, clinically significant prostate cancer; isPCa, clinically insignificant prostate cancer; fPSA, free prostate-specific antigen; tPSA, total prostate-specific antigen; PV, prostate volume; PSAD, prostate-specific antigen density; TZV, transitional zone volume; PZV, peripheral zone volume; PSADPZ, prostate-specific antigen density of peripheral zone; aPSADPZ, adjusted prostate-specific antigen density of peripheral zone; IQR, interquartile range.

### Univariate and Multivariate Regression Analyses of Independent Predictors for Diagnosing PCa and csPCa

As shown in **Table 2**, f/tPSA, PSAD, TZV, PZ ratio, and aPSADPZ were important predictors for diagnosing PCa and csPCa in univariate logistic regression analysis. Age was only important for csPCa while PV was only important for PCa. The findings of multivariate analysis are presented in **Table 3**. Notably, only aPSADPZ was included in the predictive model of PCa, while age, f/tPSA, PZ ratio, and aPSADPZ were included in the predictive model of csPCa.

**Table 2:** Univariate regression analyses for various parameters to detect PCa and csPCa in biopsy-naïve men with PIRADS v2 categories 3.

Characteristic	PCa diagnosis		csPCa diagnosis	
	OR (95% CI)	*p*	OR (95% CI)	*p*
Age (years)			1.081 (1.008–1.159)	0.028
f/tPSA	0.000 (0.000–0.237)	0.016	0.000 (0.000–0.010)	<0.01
PV (ml)	0.976 (0.957–0.996)	0.019		
PSAD (ng ml^−2^)	67.313 (3.692–1,227.389)	<0.01	168.659 (6.221–4,572.278)	<0.01
TZV (ml)	0.959 (0.930–0.988)	0.015	0.961 (0.927–0.997)	0.033
PZ ratio	204.535 (9.831–4,255.171)	<0.01	319.093 (7.446–13,675.391)	<0.01
aPSADPZ (ng ml^−2^)	29,110.383 (134.685–6,291,826.949)	<0.01	32,018.766 (112.233–9,134,606.470)	<0.01

PCa, prostate cancer; csPCa, clinically significant prostate cancer; f/tPSA, free/total prostate-specific antigen; PV, prostate volume; PSAD, prostate-specific antigen density; TZV, transitional zone volume; aPSADPZ, adjusted prostate-specific antigen density of peripheral zone; OR, odds ratio; CI, confidence interval.

**Table 3:** Multivariate regression analyses for various parameters to detect PCa and csPCa in biopsy-naïve men with PIRADS v2 categories 3.

Characteristic	PCa diagnosis		csPCa diagnosis	
	OR (95% CI)	*p*	OR (95% CI)	*p*
Age (years)			1.170 (1.074–1.275)	<0.01
f/tPSA	0.101 (0.000–24.039)	0.471	0.000 (0.000–0.236)	0.024
PV (ml)	0.998 (0.977–1.020)	0.881		
PZ ratio	21.941 (0.615–782.869)	0.090	269.406 (1.826–39,739.719)	0.028
aPSADPZ (ng ml^−2^)	610.587 (1.121–332,645.968)	0.046	4,650.212 (2.691–8,035,199.454)	0.026

PCa, prostate cancer; csPCa, clinically significant prostate cancer; PSA, prostate-specific antigen; f/tPSA, free/total prostate-specific antigen; aPSADPZ, adjusted prostate-specific antigen density of peripheral zone; OR, odds ratio; CI, confidence interval.

### ROC Curve Analysis of Predictive Factors in Comparison With aPSADPZ

**Figure 3:**
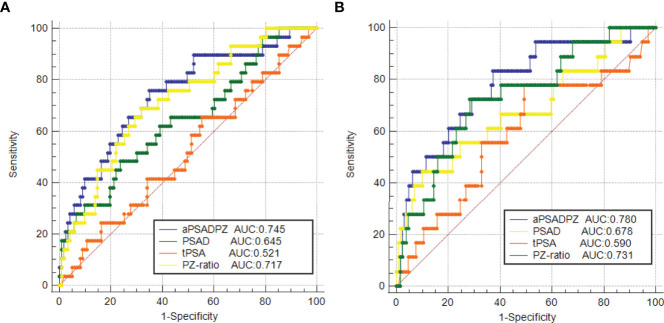
ROC curves of various parameters in the diagnosis of **(A)** PCa and **(B)** csPCa in biopsy-naïve men with PIRADS v2 categories 3. PIRADS, Prostate Imaging Reporting and Data System; tPSA, total prostate-specific antigen; PSAD, prostate-specific antigen density; aPSADPZ, adjusted prostate-specific antigen density of peripheral zone; PZ ratio, peripheral zone ratio; PCa, prostate cancer; csPCa, clinically significant prostate cancer; ROC, receiver operating characteristic; AUC, area under the curve.

ROC curve analysis revealed that the AUC for aPSADPZ in the diagnosis of PCa and csPCa was 0.745 and 0.780, respectively. Compared with other parameters, PSAD was 0.645 and 0.678, PZ ratio was 0.717 and 0.731, and PSA was 0.521 and 0.590, respectively (**Figure 3**). After pairwise comparison, the AUC of aPSADPZ was significantly larger than PSA and PSAD for PCa diagnosis (aPSADPZ vs. PSA, *Z* value: 3.488, *p* < 0.01; aPSADPZ vs. PSAD, *Z* value: 3.169, *p* < 0.01) and csPCa diagnosis (aPSADPZ vs. PSA, *Z* value: 2.440, *p* = 0.01; aPSADPZ vs. PSAD, *Z* value: 2.560, *p* = 0.01). The AUCs between aPSADPZ and PZ ratio for PCa diagnosis (aPSADPZ vs. PZ ratio, *Z* value: 0.561, *p* = 0.575) and csPCa diagnosis (aPSADPZ vs. PZ ratio, *Z* value: 0.696, *p* = 0.486) had no statistical difference.

### Nomograms and Validation of the Two Models for Diagnosing PCa and csPCa

**Figure 4:**
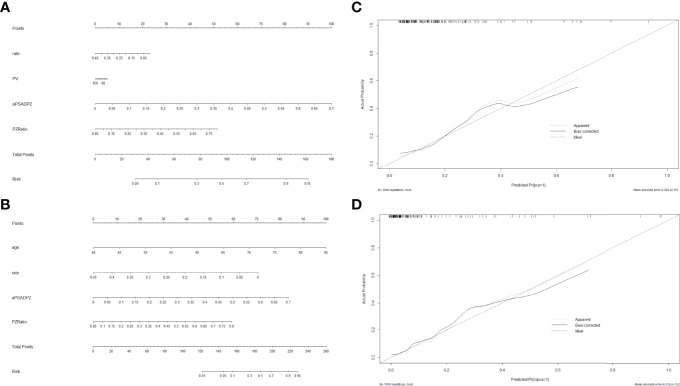
Nomogram of two models for predicting the probability of **(A)** PCa and **(B)** csPCa in biopsy-naïve men with PIRADS v2 categories 3. Calibration curves of these two nomograms in the diagnosis of **(C)** PCa and **(D)** csPCa in biopsy-naïve men with PIRADS v2 categories 3. PSA, prostate-specific antigen; aPSADPZ, adjusted prostate-specific antigen density of peripheral zone; PV, prostate volume; PIRADS, Prostate Imaging Reporting and Data System; PCa, prostate cancer; csPCa, clinically significant prostate cancer.

**Figure 5:**
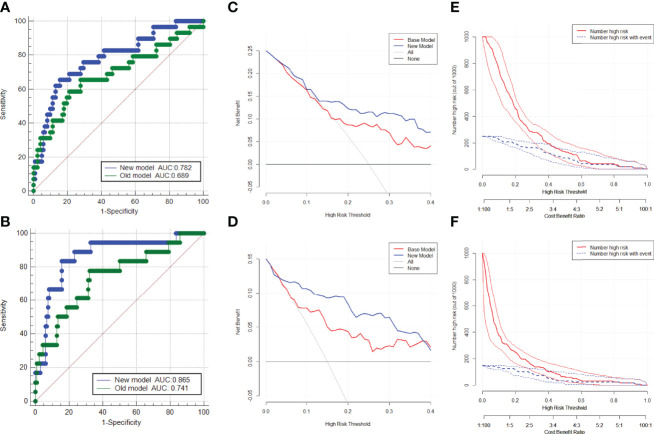
ROC curves of the two models in the diagnosis of **(A)** PCa and **(B)** csPCa in biopsy-naïve men with PIRADS v2 categories 3. Decision curve analysis of the two models for predicting the occurrence of **(C)** PCa and **(D)** csPCa in biopsy-naïve men with PIRADS v2 categories 3. Clinical impact curves of the two models for the diagnosis of **(E)** PCa and **(F)** csPCa in biopsy-naïve men with PIRADS v2 categories 3. AUC, area under the curve; ROC, receiver operating characteristic; PCa, prostate cancer; csPCa, clinically significant prostate cancer; PIRADS, Prostate Imaging Reporting and Data System.

Based on the multivariate regression coefficients, nomograms (**Figures 4A, B**) were used to visualize the predictive results. The calibration and discrimination abilities of these nomograms were further validated with an internal cohort (1,000 bootstrap resamples, **Figures 4C, D**). The C-index of the two nomograms was 0.762 and 0.880. Compared to the base model (including PSA, f/tPSA, and PSAD), the new model (including age, aPSADPZ, and PZ ratio) exhibited obviously higher AUC values (PCa: 0.782 vs. 0.689, *p* = 0.0931; csPCa: 0.865 vs. 0.741, *p* = 0.0284) for predicting csPCa (**Figures 5A, B**). Calibration curves showed excellent calibration between the actual and predicted probabilities of the new model for diagnosing PCa and csPCa.

### Decision Curve Analysis for Diagnosing PCa and csPCa

The DCA indicated that the net benefit of the new model was better than that of the base model for predicting PCa and csPCa in the defined regions of interest (10%–40% probability) (**Figures 5C, D**). In addition, clinical impact curves showed that in the high-risk threshold, the number of patients with PCa and csPCa predicted by the new model was in good agreement with the actual number of patients with PCa and csPCa (**Figures 5E, F**).

## Discussion

We believed that both PSAD and PZ ratio are dependent on the correct measurement of PV and TZV. Colvin et al. (13) reported the current PIRADS V2.1 recommendation of either traditional ellipsoid or segmentation volume measurements as viable methods to assess prostate volume. The predictive capability of PSAD for csPCa was not significantly different between the two measurements. Stanzione et al. (14) also made a similar viewpoint. Thus, we still used the traditional ellipsoid volume measurement to calculate PV and TZV. Luis et al. (15) showed that PSAD can improve the early detection of csPCa. Porcaro et al. (16) found that increased prostate volume index (PVI), which is defined as the ratio of the volume of the TZV to the PZV, decreased the risk of increased tumor load and was associated with less aggressive PCa biology in patients at baseline random biopsies. They made a point that PVI is a pure measurement that is separate from PSAD, which compared the PSA serum levels with the PV and is largely influenced by TZV. Chang et al. (17 *)* showed that the PZ ratio could be used as a predictor of PCa. We believe that PVI and PZ ratio have the same diagnostic efficacy because they both represent the percentage of the PZV in the PV. Thus, we only chose PZ ratio for PCa prediction. Koo et al. (18) and Lee et al. (19) indicated that PZPSAD was better than PSAD for the detection of PCa. Schneider et al. (20) found that TZPSAD exhibited a stronger correlation to cancer aggressiveness compared to PSAD. In our views, since BPH is mainly due to hyperplasia in the TZ, while PCa predominantly occurs in the PZ (21), conventional PSAD does not take into account whether the hyperplastic prostate tissue is mainly in the TZ or the PZ. Recently, the PIRADS score has shown important clinical significance in PCa diagnosis (6). The current consensus is that PIRADS 3 is a gray area for the diagnosis of PCa (22). The lesions of PIRADS 3 do not have typical features (7). Satoshi et al. (23) demonstrated that PSAD was useful for the diagnosis of PCa in men with a PIRADS score ≤ 3, thus avoiding unnecessary biopsy. Gaudiano et al. (24) found that PSAD and location within the prostate gland are associated with an increased risk of the presence of PCa in patients with PIRADS 3. In our study, we combined PSAD with PZ ratio to obtain a new index (aPSADPZ), which achieved a better predictive outcome. We found that aPSADPZ had an advantage over PSA and PSAD in the diagnosis of PCa and csPCa in biopsy-naïve men with PIRADS 3. In our opinion, PCa is mostly found in the PZ (21), and tumors in the PZ possibly increase the levels of PSA in patients with a high PZ ratio. Our results showed that the aPSADPZ had a significant predictive ability in both univariate and multivariate analyses, indicating that it was the best predictor of PCa and csPCa in this study.

Eastham et al. (25) reported the first nomogram to predict PCa in 1999. Zhang et al. (26) reported that a model including PIRADS, PSAD, and age showed internally validated high discrimination and calibration for the absence of PCa and csPCa in biopsy-naïve men with PIRADS ≤ 3. To our knowledge, this is a new nomogram that combines aPSADPZ, PZ ratio, and age to predict PCa in biopsy-naïve men with PIRADS 3. In our study, the new model’s C-index of csPCa diagnosis was 0.88, which had good diagnostic performance. The AUC of the new model was 0.865 for csPCa diagnosis, whereas the base model’s AUC was 0.741. The new model had a significantly higher diagnostic efficiency compared to the base model (0.865 vs. 0.741, *p* = 0.0284). Further validation of the new model for diagnosing csPCa indicated its excellent performance. In the defined region of interest (10%–40% probability), which means difficulty in making clinical decisions, the net benefit of the new model is higher than that of the base model in the DCA. It means that when the probability of csPCa is moderate or low, which makes it difficult to make a clinical decision as to whether to biopsy or not, the new model can increase the rate of detection for csPCa and avoid unnecessary biopsy. The new model is more suitable for guiding clinical decision-making in men with PIRADS 3. On the other hand, we found a good calibration between the actual and predicted probabilities of the new model in the region of high probability. Taken together, for low or medium probability populations, the new model can detect more csPCa and reduce unnecessary biopsies, while for high probability populations, it will not miss csPCa and cause adverse outcomes.

Our study has several limitations: (1) This was a retrospective study performed at a single institution with a possible risk of selection bias. (2) PIRADS scores are dependent on the experience of the radiologist, and may vary from physician to physician. (3) The definition of csPCa used in this study does not include all clinically significant diseases, in that ISUP GG1 with a high tumor volume load may be significant and ISUP GG2 with a low tumor volume load may be insignificant. (4) We did not consider an upgrade or a downgrade of Gleason scores in specimens after radical prostatectomy, which may be a limitation for the determination of csPCa.

## Conclusions

In summary, aPSADPZ has a higher predictive accuracy for the diagnosis of PCa and csPCa in biopsy-naïve men with PIRADS 3 than the conventional indicators, which may decrease the risk of misdiagnosis and reduce the number of unnecessary biopsies. The prediction model of aPSADPZ, PZ ratio, and age can improve csPCa detection, increase diagnostic accuracy, and avoid unnecessary biopsies.

## Data Availability Statement

The raw data supporting the conclusions of this article will be made available by the authors, without undue reservation.

## Ethics Statement

The studies involving human participants were reviewed and approved by the Institutional review board of The First Affiliated Hospital of Soochow University. The patients/participants provided their written informed consent to participate in this study.

## Author Contributions

CH helped in project development and data analysis, and wrote the manuscript. FQ was involved in project development and data collection, and wrote the manuscript. DJ was involved in project development and data analysis and wrote the manuscript. JP and XW helped in data analysis. ZC performed the statistical analysis. XW helped in the drafting of the manuscript. XZ, LG, and JH helped in data collection. YH helped in project development and edited the manuscript. All authors contributed to the article and approved the submitted version.

## Funding

This work was supported by two grants from the Key Research and Development Program of Jiangsu Province (No. BE2020654 and No. BE2020655) and a grant from the General Program of Jiangsu Health Commission (No. H2019040).

## Conflict of Interest

The authors declare that the research was conducted in the absence of any commercial or financial relationships that could be construed as a potential conflict of interest.

## Publisher’s Note

All claims expressed in this article are solely those of the authors and do not necessarily represent those of their affiliated organizations, or those of the publisher, the editors and the reviewers. Any product that may be evaluated in this article, or claim that may be made by its manufacturer, is not guaranteed or endorsed by the publisher.
