# Supplementary material for: New Diagnostic Model for Clinically Significant Prostate Cancer in Biopsy-Naïve Men With PIRADS 3
Source: Front Oncol. 2022 Jul 4;12:908956. doi: 10.3389/fonc.2022.908956 (PMC9289138; doi:10.3389/fonc.2022.908956)
Supplement: Supplementary file 1 [file Table_1.docx]

**Supplementary table 1. Imaging protocol for prostatic mpMRI**

| Sequence | Imaging protocol |
| --- | --- |
| T1WI | repetition time(msec)/echo time(msec), 700/14;  section thickness, 3.5 mm;  intersection gap,0.5 mm;  ﬁeld of view, 25 cm;  matrix, 384 × 336 |
| T2WI | repetition time(msec) / effective echo time(msec), 3500–6980/105;  section thickness, 3.5 mm;  ﬁeld of view, 20–25 cm;  matrix,384 × 384 |
| DWI | repetition time(msec) / echo time(msec),5000/72;  ﬁeld of view, 20 cm;  matrix, 128 × 128;  section thickness,3.5 mm;  a parallel imaging factor of 2  using b values of 0,100,800,1000, and 1500 s/mm2. |
| ADC | measured by using a mono-exponential model. |
| DCE | repetition time(msec) / echo time(msec), 4.2/1.34;  ﬂip angle,12 ;  ﬁeld of view, 24 cm;  matrix, 224 × 224;  slice thickness, 3 mm;  a parallel imaging factor of 2.  After two acquisitions, an intravenous contrast agent (Medtron AG, Saarbruecken, Germany) was administered at 1ml kg-1body weight and 2.5 ml s-1 injection rate through a 20G antecubital intravenous line. Bolus injection was performed with an MR-compatible power injector (Spectris; Medrad, Pittsburgh, PA) followed by a 15-mL saline ﬂush. The DCE imaging was continued for 5.0 minutes after the contrast medium injection. |

DWI: diffusion-weighted imaging, ADC: apparent diffusion coefficient,DCE: dynamic contrast enhanced.
